# Dietary Corn Bran Fermented by *Bacillus subtilis* MA139 Decreased Gut Cellulolytic Bacteria and Microbiota Diversity in Finishing Pigs

**DOI:** 10.3389/fcimb.2017.00526

**Published:** 2017-12-22

**Authors:** Ping Liu, Jinbiao Zhao, Pingting Guo, Wenqing Lu, Zhengying Geng, Crystal L. Levesque, Lee J. Johnston, Chunlin Wang, Ling Liu, Jie Zhang, Ning Ma, Shiyan Qiao, Xi Ma

**Affiliations:** ^1^State Key Laboratory of Animal Nutrition, College of Animal Science and Technology, China Agricultural University, Beijing, China; ^2^Department of Animal Sciences, South Dakota State University, Brookings, SD, United States; ^3^Swine Nutrition and Production, West Central Research and Outreach Center, University of Minnesota, Morris, MN, United States; ^4^Department of Animal Husbandry and Veterinary Medicine, Beijing Vocational College of Agriculture, Beijing, China; ^5^Department of Internal Medicine, Department of Biochemistry, University of Texas Southwestern Medical Center, Dallas, TX, United States

**Keywords:** *Bacillus subtilis*, cellulolytic bacteria, corn bran, dietary fiber, fermentation, gut microbiota

## Abstract

Solid-state fermentation of feedstuffs by *Bacillus subtilis* MA139 can reduce insoluble dietary fiber content *in vitro* and improve growth performance in pigs. This study was conducted to investigate the effects of dietary corn bran (CB) fermented by *B. subtilis* on growth performance and gut microbiota composition in finishing pigs. A total of 60 finishing pigs were allocated to 3 dietary treatments consisting of a control (CON) diet, a 10% CB diet, and a 10% fermented CB (FCB) diet in a 21 d feeding trial. Growth performance and nutrient digestibility were evaluated. Fecal samples were determined for bacterial community diversity by 16S rRNA gene amplicon sequencing. The dietary CB and FCB did not affect growth performance of finishing pigs. The digestibility of organic matter was decreased in both CB and FCB treatments compared with CON group (*P* < 0.05). The α-diversity for bacterial community analysis of Chao 1 in FCB treatment was lower than CON treatment (*P* < 0.05). The *Fibrobacteres* phylum belongs to cellulolytic bacteria was isolated, and their relative abundance in CB group showed no difference between CON and FCB treatments. The abundance of *Lachnospiraceae_NK4A136_group* in CB treatment was higher than CON and FCB groups (*P* < 0.05), whereas the population of *norank_f_Prevotellaceae* was higher in FCB group compared to CON and CB groups (*P* < 0.05). In conclusion, dietary FCB decreased the abundance of bacterial communities, particularly the population of bacteria related to cellulolytic degradation.

## Introduction

The consumption of dietary fiber plays an essential role in maintaining homeostasis of the gut ecosystem. Bacterial metabolites and prebiotics from dietary fiber are involved in various physiological processes for modulating health in humans and animals (Desai et al., 2016; Martens, 2016). Low fiber intake often results in chronic diseases related to nutrient metabolism and gut inflammatory disorders, such as obesity, diabetes, and inflammatory bowel diseases (Han et al., 2017; Ma et al., 2017). Thus, there is an increasing interest focused on the link between different fibrous ingredients and gut health. High fiber diets improved intestinal morphology, mucin secretion, and growth of beneficial bacteria (Hedemann et al., 2009; Chen H. et al., 2015; Tian et al., 2017). This has been attributed to the role of short-chain fatty acids (SCFAs) in the gut health and they are end-products of dietary fiber fermented by gut microbiota (Chen J. et al., 2015). The major SCFAs are acetate, propionate, and butyrate, they serve as energy substrates for the colonic epithelium and modulate the immune system (Fan et al., 2015; Koh et al., 2016). The effects of high fiber on growth performance in pigs have been studied. Some reports indicated that fiber-rich diets increased weight gain (Bertram et al., 2009; Gerritsen et al., 2012), while other researchers showed reduced growth or no influence on growth performance accompanied by reduced digestibility of nutrients and energy (Jaworski et al., 2017; Morowitz et al., 2017).

The milling of corn for food constituents results in a large number of co-products. Corn bran (CB) is a common fibrous corn by-product that has been widely utilized in animal feed due to its relatively lower cost. CB primarily consists of insoluble fiber with ~280 g/kg cellulose and 700 g/kg hemicellulose. This indigestible corn bran contains many bioactive components, such as corn fiber gum, cellulosic fiber gel, and xylo-oligosaccharides (Rose et al., 2010). Previous studies have shown that arabinoxylan and cellulose were the major components extracted from wheat bran, which exerted positive effects on intestinal barrier function and increased the population of *Lactobacillus* in piglets (Chen H. et al., 2015; Hashemi et al., 2017). In contrast, information about how CB impacts the gut bacterial community is very limited.

To harvest the production of useful bioactive components from corn barn, different processing technologies have been used. Fermentation technology has been applied for modification of biological materials into useful products and for reducing the insoluble fiber content of feed (Pallin et al., 2016). *Bacillus subtilis* MA139 was identified in previous study having high antimicrobial activity against *Escherichia coli, Salmonella typhimurium*, and *Staphylococcus aureus* (Yang et al., 2015). Diets that contained *B. subtilis* MA139 improved daily gain and feed conversion in weaned pigs, and *B. subtilis* was recommended as a potential alterative to in-feed antibiotics (Horita et al., 2015). *B. subtilis* can also produce various carbohydrase enzymes for polysaccharide degradation (Rhee et al., 2014). *B. subtilis* MA139 co-inoculated with lactic acid bacteria were applied with solid-state fermentation for the production of fermented wheat bran and soybean bran feedstuffs (Vijayaraghavan et al., 2016). However, the effect of dietary CB fermented by *B. subtilis* in pigs has not been reported. It is important to understand how complex polysaccharides influence gut microbiota in humans and monogastric animals. Therefore, the objective of the present study was to compare the effects of diets that contained CB or fermented CB (FCB) on growth performance, nutrient digestibility, and gut microbiota composition in finishing pigs.

## Materials and methods

### Preparation of fermented CB

This study was carried out in accordance with the recommendations of “Laboratory Animals-Guideline of Welfare and Ethics of China (ICS 65.020.30), the Institutional Animal Care and Use Committee of China Agricultural University.” The protocol was approved by the Institutional Animal Care and Use Committee of China Agricultural University.

Corn bran was purchased from Wellhope Agri-tech Co. Ltd. (Beijing, China). *Bacillus subtilis* MA139 was provided by Ministry of Agriculture Feed Industry Centre on September 5, 2016 (Beijing, China), and the *Enterococcus faecium* and *Saccharomyces cerevisae* were purchased from Yaxin Biotechnology Co. Ltd. (Taiwan, China). *Bacillus subtilis* MA139 and *S. cerevisae* were cultured aerobically in mixed nutrient broth (MNB) and yeast peptone dextrose (YDP) by shaking at 250 × g for 24 h. *E. faecium* was grown in MRS (de Man, Rogosa and Sharpe) broth in 5% CO_2_ incubator at 37°C for 22 h as previous report (Vijayaraghavan et al., 2016). The unsterilized CB was mixed with the starter cultures including *B. subtilis* MA139, *E. faecium*, and *S. cerevisae* in a ratio of 10:1 (w/v). After mixing, the treated CB contained 5.7 × 10^7^ cfu/g *B. subtilis* MA139, 2.6 × 10^7^ cfu/g *E. faecium*, and 1.5 × 10^7^ cfu/g *S. cerevisae*. The CB was packed and sealed in a polythene bag with a capacity of 25 kg (Rou Duoduo Biotechnology Co., Beijing, China) at 30°C for 14 d. The bag was equipped with a one-way value to release gas produced during the fermentation. At the end of the solid-state fermentation, the treated CB contained 0.27 mg/g butyrate and 0.93 mg/g lactate at pH 6.2 analyzed by high performance ion chromatography and pH meter. The final moisture content of FCB was ~30% according to method 930.15 (AOAC International, 2007). Samples of CB and FCB were collected for chemical analysis, including starch, crude protein, cellulose, hemicellulose, lignin, soluble dietary fiber, insoluble dietary fiber, and non-starch polysaccharide.

### Animals, diet treatments, and sampling

Finishing pigs [*n* = 60, Duroc × (Landrance × Yorkshire), 135 ± 1 d age, 65.73 ± 0.7 kg body weight] were allocated randomly to 3 groups balancing for litter and gender. Pigs were housed in commercial flat-deck pens (4 pigs per pen, 5 pens per treatment). Room temperature was maintained at 22°C, and the humidity was maintained constant at 65~75%. Water and feed were provided *ad libitum*. Twenty pigs in each group received one of 3 experimental diets (Supplemental Table 1) based on corn and soybean meal included the control (CON) diet, a 10% corn barn (CB) diet, and a 10% fermented corn barn (FCB) diet. Seven days of acclimation to diets and pens were allowed before the trial started. The experiment lasted for 21 d.

The amount of feed provided for pigs was recorded and unconsumed feed was weighed daily for the determination of average daily feed intake (ADFI) (Jaworski et al., 2017). Pigs were weighed individually on day 0 and 21. Average daily gain (ADG) was calculated as weight gain (final body weight—initial body weight) divided by the number of treatment days. Feed conversion ratio (FCR) was calculated as the ratio of ADFI and ADG (Jaworski et al., 2017). A sample of each of the experimental diets was collected for chemical analysis. A fecal sample (~300 g) was collected on d 18, 19, and 20 from 1 pig per pen (*n* = 5 pigs per treatment) and pooled prior to drying. Feed and fecal samples were oven-dried at 65°C for 72 h. All samples were ground to pass through 40-mesh sieve for nutrient digestibility analysis.

On 21 d, fresh fecal samples from at least 1 pig per pen (*n* = 7 pigs per treatment) were collected in sterile tubes, immediately snap-frozen in liquid nitrogen, and stored at −80°C for subsequent analysis of gut microbiota composition and concentrations of organic acids.

### Chemical analysis

Samples of diets and feces were analyzed for gross energy (GE), dry matter (DM), organic matter (OM), crude protein (CP), ether extract (EE), and total dietary fiber (TDF) according to the AOAC procedure. Gross energy was determined by automatic adiabatic oxygen bomb calorimetry (Parr 1281, Automatic Energy Analyzer, Moline, IL, USA). Acid insoluble ash (AIA) in the diets and feces was measured (De Coca-Sinova et al., 2011). Apparent total tract digestibility of GE, DM, OM, CP, EE, and TDF were determined (Medel et al., 1999).

Insoluble and soluble dietary fiber content of CB and FCB were analyzed according to method 991.43 (AOAC International, 2007) using the Ankom TDF Dietary Fiber Analyzer (Ankom Technology, USA). Cellulose, hemicellulose, and lignin content of ingredients were measured (Bertram et al., 2009). Non-starch polysaccharides and their monosaccharide components were measured on the basis of alditol acetates by gas-liquid chromatography (Aglilent GC 6890, USA) with a column of 30 m × 0.25 mm × 0.25 mm (Agilent DB-225, USA) at speed of 20 mL/min. The column temperature was 220°C and the injector and detector temperatures were 250°C.

### Extraction of nucleic acids and PCR amplification

Microbial DNA was extracted from fecal samples using the DNA Kit (Omega Bio-tek, Norcross, GA, USA) according to manufacturer's protocols. The V4-V5 region of the bacteria 16S ribosomal RNA gene were amplified by PCR (95°C for 2 min, followed by 25 cycles at 95°C for 30 s, 55°C for 30 s, and 72°C for 30 s and a final extension at 72°C for 5 min) using primers 515F 5′-barcode- GTGCCAGCMGCCGCGG)-3′ and 907R 5′-CCGTCAATTCMTTTRAGTTT-3′ (Tsai et al., 2011), where barcode is an eight-base sequence unique to each sample. PCR reactions were performed in triplicate in 20 μL mixture containing 4 μL of 5 × FastPfu Buffer, 2 μL of 2.5 mM dNTPs, 0.8 μL of each primer (5 μM), 0.4 μL of FastPfu Polymerase, and 10 ng of template DNA.

### Illumina sequencing and data analysis

Amplicons were extracted from 2% agarose gels and purified using the AxyPrep DNA Gel Extraction Kit (Axygen Biosciences, Union City, CA, USA) and quantified using QuantiFluor™-ST (Promega, USA). Purified amplicons were pooled in equimolar concentrations and paired-end sequenced (2 × 300) on an Illumina MiSeq platform according to the standard protocols. Raw fastq files were demultiplexed, and quality-filtered using QIIME (version 1.17). Operational taxonomic units (OTUs) were clustered with 97% similarity cutoff using UPARSE and chimeric sequences were identified and removed using UCHIME. The taxonomy of each 16S rRNA gene sequence was analyzed by RDP Classifier (http://rdp.cme.msu.edu/) against the silva (SSU128) 16S rRNA database using confidence threshold of 70%. Raw data were submitted to NCBI sequence read archive database with the accession No. SRP105389.

### Quantification of SCFAs in fecal samples

Fecal sample (0.5 g) was weighed into a 10 mL polypropylene tube and 8 mL deionized water was added. After using an ultrasonic bath for 30 min, the mixture was centrifuged at 8,000 × g for 10 min. The suspension was diluted (1:50) with water and filtered through a 0.22 μm filter. A 25 μL sample solution was extracted and analyzed for the SCFAs, including lactate, acetate, propionate, isobutyrate, isovalerate, and valerate by a high performance ion chromatography (ICS-3000 Dionex, USA) (Hammami et al., 2015). These SCFAs were separated by an AS11 analytical column (250 × 4 mm) and an AG11 guard column under the gradient condition: 0–5 min, 0.8–1.5 mM; 5–10 min, 1.5–2.5 mM, 10–15 min, 2.5 mM, the flow rate was 1.0 mL/min. The gradient was carried out with potassium hydroxide.

### Statistical analysis

Data on growth performance, nutrient digestibility, and fecal organic acid content was performed using SPSS 19.0 (Chicago, IL, USA). The data were analyzed using one-way analysis of variance (ANOVA) followed by Tukey's test, and the results were presented as mean values ± SEM. Microbiota diversity metrics were performed from normalized OTU reads using R software (version 3.2.2) (https://www.r-project.org/). The relative abundance of gut microbiota composition (phyla, classes, orders, families, and genera) was analyzed by the Kruskal–Wallis method. Differences were considered significant at *P* < 0.05.

## Results

### The chemical composition of CB and FCB

Corn bran mainly contained insoluble dietary fiber (Table 1). The contents of cellulose, hemicellulose, and lignin were ~23% lower in FCB than CB; while soluble dietary fiber and non-starch polysaccharide contents were increased ~53 and 29%, respectively. The dominant monosaccharide residues of non-starch polysaccharide in CB and FCB were arabinose, xylose, and glucose. The contents of arabinose, xylose, and glucose in FCB were increased ~50, 39, and 23%, respectively.

**Table 1:** Composition of corn bran (CB) and fermented CB (FCB) (g/kg, DM basis)^1^.

**Items**	**CB**	**FCB**
Starch	243.2	131.3
Organic matter	971.7	978.4
Crude protein	164.8	115.1
Cellulose	120.1	93.1
Hemicellulose	332.7	262.2
Lignin	22.9	11.5
Total dietary fiber	601.3	694.4
SDF	66.7	102.6
IDF	534.7	591.8
SDF/IDF	12.5	17.3
NSP	535.5	689.7
Rhamnose	3.5	2.4
Fructose	2.0	2.9
Ribose	3.0	2.7
Arabinose	89.5	134.2
Xylose	148.0	206.0
Mannose	18.5	15.1
Galactose	24.5	23.3
Glucose	246.5	303.1

1 *SDF, soluble dietary fiber; IDF, insoluble dietary fiber; NSP, non-starch polysaccharides*.

### Growth performance and nutrient digestibility

There were no differences in ADFI, ADG, and FCR among the dietary treatments (Table 2), which indicated that there are no effect on growth performance when treated by dietary CB and FCB. The apparent digestibility of GE, DM, CP, EE, and TDF in different groups were detected (Table 3). The apparent digestibility of OM decreased (*P* < 0.05) in CB and FCB treatments compared to CON group. There was no effect of dietary treatment on digestibility of other measured nutrients.

**Table 2:** Effects of dietary corn bran (CB) and fermented CB (FCB) on growth performance of finishing pigs^1^.

**Items**	**Dietary treatments**			
	**CON**	**CB**	**FCB**	***P*-value**
ADFI, kg	2.44 ± 0.19	2.60 ± 0.13	2.51 ± 0.09	0.751
ADG, g	852 ± 64.7	897 ± 25.6	856 ± 27.0	0.726
FCR	2.88 ± 0.15	2.92 ± 0.14	2.99 ± 0.10	0.844

1 *Twenty pigs per treatment (5 pens of 4 pigs) were measured for growth performance in a 21 d feeding trial. The results were presented as mean values ± SEMs. Data were analyzed by one-way ANOVA with Tukey's test. CON, control group; ADFI, average daily feed intake; ADG, average daily gain; FCR, feed conversion ratio*.

**Table 3:** Effect of dietary corn bran (CB) and fermented CB (FCB) on nutrient digestibility of finishing pigs^1^.

**Items, %**	**Dietary treatments**			
	**CON**	**CB**	**FCB**	***P*-value**
GE	88.58 ± 0.53	87.43 ± 0.42	87.68 ± 0.16	0.194
DM	89.46 ± 0.49	88.27 ± 0.37	88.50 ± 0.12	0.123
OM	90.91 ± 0.44^a^	89.40 ± 0.40^b^	89.70 ± 0.12^b^	0.039
CP	86.73 ± 0.64	86.40 ± 0.51	87.85 ± 0.28	0.085
EE	64.05 ± 1.51	67.17 ± 1.32	65.94 ± 1.22	0.188
TDF	62.12 ± 1.43	63.87 ± 0.94	64.75 ± 1.38	0.203

1 *Fecal samples from 1 pig per pen (n = 5 pigs per treatment) performed for nutrient digestibility in a 21 d feeding trial. The results were presented as mean values ± SEMs. Data were analyzed by one-way ANOVA with Tukey's test. Different superscript lowercase letters within each row mean significantly different (P < 0.05). CON, control group; GE, gross energy; DM, dry matter; OM, organic matter; CP, crude protein; EE, ether extract; TDF, total dietary fiber*.

### Bacterial community richness and biodiversity

**Figure 1:**
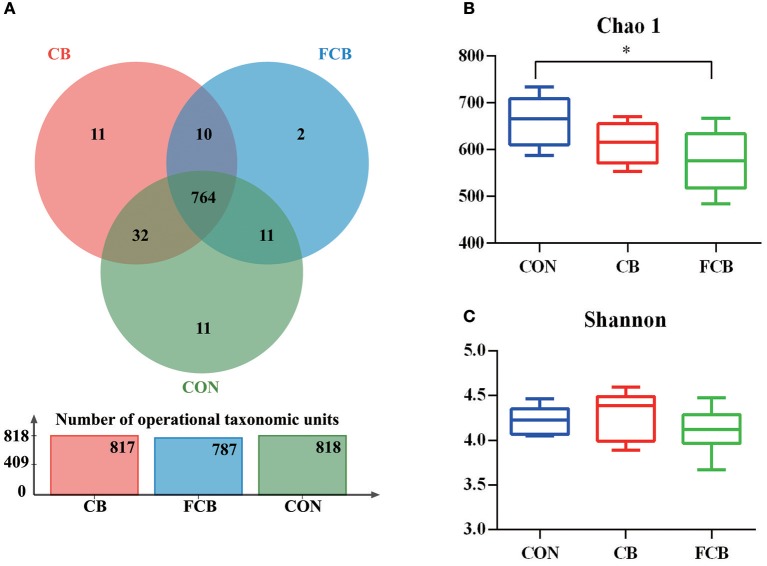
Effect of dietary corn bran (CB) and fermented CB (FCB) on intestinal microbiota richness in finishing pigs. **(A)** OTU Venn of 3 dietary treatments. **(B)** The Chao 1 index of bacterial community. **(C)** Shannon index of bacterial community. Fecal samples from 7 pigs per treatment were performed for 16S rRNA gene amplicon sequencing analysis in a 21 d feeding trial. The results were analyzed by Kruskal–Wallis H test and presented as mean values of different bacteria, and asterisk means *P* < 0.05. CON, control group.

There were 818, 817, and 787 operational taxonomic units (OTUs) obtained from pigs fed CON, CB, and FCB dietary treatments, respectively, of which 764 were common OTUs among 3 experimental groups (Figure 1A). Moreover, a total of 24 unique OTUs were found within CON, CB, and FCB groups (11, 11, and 2, respectively). The α-diversity index of Chao 1 in the FCB group was lower compared to the CON group (*P* < 0.05), and the Shannon index was not affected by dietary treatments (Figures 1B,C).

**Figure 2:**
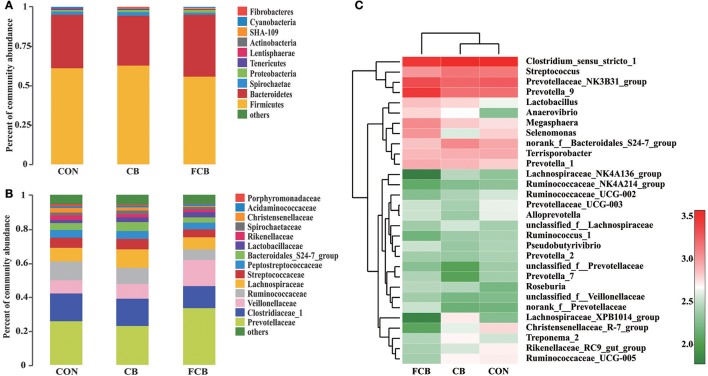
Effect of dietary corn bran (CB) and fermented CB (FCB) on gut microbiota composition in finishing pigs. **(A)** Microbial community bar plot at the phylum level with the relative abundance higher than 0.05%. **(B)** Microbial community bar plot at the family level with the relative abundance higher than 1%. **(C)** Microbial community heat map of the top 30 bacteria at the genus level. Fecal samples from 7 pigs per treatment were performed for 16S rRNA gene amplicon sequencing analysis in a 21 d feeding trial. The results were analyzed by Kruskal–Wallis H test and presented as mean percentage of different bacteria. CON, control group.

Fifteen distinct phylum were identified in feces of finishing pigs fed different CB supplementations (Supplemental Table 2), where *Firmicutes* and *Bacteroidetes* accounted for ~94% within all dietary treatments (Figure 2A). The study detected Fibrobacteres phylum, which is associated with cellulolytic bacteria, and there was no difference between treatments where relative abundance in CON, CB, and FCB groups was 0.04, 0.13, and 0.09%, respectively (Supplemental Table 2). The dominant families within the *Firmicutes* phylum consisted of *Clostridiaceae_1, Veillonellaceae, Ruminococcaceae, Lachnospiraceae, Streptococcaceae*, and *Peptostreptococcaceae*, while the main families within the *Bacteridetes* phylum were *Prevotellaceae, Bacteroidales_S24-7_group*, and *Rikenellaceae* (Figure 2B). The relative abundance of bacterial communities at the family level did not differ among experimental groups (Supplemental Table 3). The relative abundance of the top 30 genera bacteria community were analyzed (Figure 2C), and their relative abundance were higher than 1% at least 1 treatment group (Supplemental Table 4). The population of *Lachnospiraceae_NK4A136_group* in CB treatment (1.29%) was higher than CON (0.92%) and FCB (0.26%) groups (*P* < 0.05). The relative abundance of *norank_f_Prevotellaceae* was higher in FCB group (1.47%) compared to CON (0.69%) and CB (0.64%) groups (*P* < 0.05). In addition, pigs fed CON diet (2.24%) had higher abundance of *Rikenellaceae_RC9_gut_group* compared to CB (1.61%) and FCB (1.17%) treatments (*P* < 0.05).

### SCFAs in fecal samples

Lactic acid was not detected in the majority of fecal samples, and the SCFAs including acetate, priopinate, isobutyrate, butyrate, isovalerate, and valerate were not affected by the CB or FCB dietary treatments (Table 4), which suggested that SCFAs, the microbial metabolites, may not mediate the effects of dietary CB or FCB.

**Table 4:** Effect of dietary corn bran (CB) and fermented CB (FCB) on short-chain fatty acids concentrations in feces of finishing pigs^1^.

**Item, mg/g**	**Dietary treatments**			
	**CON**	**CB**	**FCB**	***P*-value**
Acetate	4.07 ± 0.17	4.26 ± 0.18	3.90 ± 0.10	0.289
Propionate	2.43 ± 0.17	2.60 ± 0.16	2.50 ± 0.09	0.682
Isobutyrate	0.35 ± 0.06	0.26 ± 0.02	0.25 ± 0.02	0.185
Butyrate	2.00 ± 0.22	1.68 ± 0.16	1.62 ± 0.09	0.259
Isovalerate	0.23 ± 0.04	0.20 ± 0.01	0.19 ± 0.01	0.352
Valerate	0.52 ± 0.08	0.41 ± 0.04	0.46 ± 0.04	0.388
Total SCFAs	9.05 ± 0.52	9.51 ± 0.48	8.84 ± 0.16	0.493

1 *Fecal samples from at least 1 pig per pen (n = 7 pigs per treatment) were performed for SCFAs analysis in a 21 d feeding trial, data were presented as mean values ± SEMs. Data were analyzed by one-way ANOVA with Tukey's test. CON, control group; SCFAs, short-chain fatty acids*.

## Discussion

This study was performed to compare the effect of CB and FCB inclusion on growth performance, nutrient digestibility, and gut microbiota composition in finishing pigs. Dietary fiber is involved in various physiological processes and plays an essential role in enhancing immune regulation and gut health (Koh et al., 2016). Corn bran is the main by-products from the corn milling process, and is commonly utilized as a feed ingredient for animals. The majority of plant polysaccharides like CB cannot be digested directly by enzymes in humans and monogastric animals, but they can be degraded by colonic bacterial communities (Sonnenburg and Sonnenburg, 2014).

Polysaccharide utilization and fiber digestibility can be improved with special processing technology. Solid-state fermentation of feedstuffs has been applied for the purpose of modifying biological materials to reduce the insoluble fiber content of feeds resulting in useful feedstuffs (Pallin et al., 2016), because the fibrolytic enzymes produced from bacteria, such as cellulases and hemicellulases, are able to degrade complex polysaccharides (Kracher et al., 2016). In this study, the contents of cellulose and hemicellulose were reduced in the FCB. Meanwhile, the level of soluble dietary fiber increased after *in vitro* fermentation compared with CB. Chemical analysis demonstrated that the solid-state fermentation makes the insoluble glycans in corn bran easily digestible. In addition, 3 monosaccharide residues (arabinose, xylose, and glucose) were increased, and these should be closely associated with the reaction of fermentation by the mixed cultures of *Bacillus subtilis* MA139, *E. faecium*, and *S. cerevisae*. It has been reported that arabinose and xylose are major sugars in the plant cell wall of wheat bran and CB (Rose et al., 2010; Chen H. et al., 2015); *B. subtilis* produce xylanases, which are able to degrade soluble and insoluble xylan substrates to acidic xylo-oligosaccharides (Rhee et al., 2014). The strain of *B. subtilis* MA139 used in this study was identified and has been used as a potential probiotic to improve pig performance (Yang et al., 2015). Further, we found an innovative strategy to apply *B. subtilis* MA139 with lactic acid bacteria to produce solid-state fermentation of feedstuffs. These co-inoculating strains showed high antimicrobial activity against on *Enterobacteriaceae*, such as *E. coli* and *S. typhimurium*. Lactic acid bacteria, such as *Lactobacillus reuteri* and *Lactobacillus fermentium*, have been applied for feed fermentation due to their beneficial effects on preservation of feeds, favorable aroma, and flavor of bacterial end-products (Pallin et al., 2016). In the present study, *E. faecium* was applied in solid-state fermentation in CB because it can synthesize enzymes to degrade starch and the bacterial metabolite of lactic acid is beneficial for modulating immune response of the host (Siepert et al., 2014; Starke et al., 2015).

Dietary fiber plays an important role in maintaining bacterial diversity and intestinal homeostasis (Chen J. et al., 2015). It is reported that high concentrations of dietary fiber result in body weight loss (Kovatcheva-Datchary et al., 2015), which may be attributed to an improvement in satiety due to fiber viscosity. Fiber-rich diets may affect satiety through an increase in mastication or changes in gut hormones levels, such as glucagon-like peptide 1 (Näslund and Hellström, 2007). A previous study showed CB resulted in a lesser desire for food intake in humans (Rose et al., 2010). In the present study, the pigs fed CB or FCB treatments did not show significant differences in daily feed intake and average daily gain and digestibility of organic matter decreased in both CB and FCB treatments compared to CON group. This observation was in agreement with the previous studies in pigs (Bindelle et al., 2008; Bertram et al., 2009). The result suggests that diets containing 10% CB or FCB did not affect satiety response in finishing pigs over 21 d study.

The gut bacterial community and the abundance of bacterial metabolites are influenced by diets, in turn, the gut microbiota composition impacts nutrient digestibility (Fan et al., 2015, 2017a). Dietary fiber is characterized by different monosaccharides within complex polymers of ß-1,4-linked units of hexose (cellobiose and glucose) and/or pentose (arabinose and xylose) sugars (Martin et al., 1998), which cannot be digested in the small intestine of monogastric animals due to a lack of cellulolytic enzymes. However, the most dense and diverse bacterial community inhabits the large intestine and can produce a range of cellulolytic enzymes to degrade different fiber component in diet. Consequently, the activity of bacterial physiology relates to the fiber metabolism in the gut. The degradation of complex carbohydrates requires a range of bacterial enzymatic activities, normally accomplished by a consortium of bacteria rather than a single species (Scott et al., 2008).

There is a considerable degree in variation of the intestinal microbiota composition between studies, but *Firmicutes* and *Bacteroidetes* were the most dominant phyla in healthy individuals (Flint et al., 2007). In our study, the *Firmicutes* and *Bacteriodetes* were numerically dominant in finishing pigs with the abundance higher than 94%, and the result is consistent with fecal microbiota from pigs reported in other studies observed (Heinritz et al., 2016). It has been reported that fiber-rich diets result in increased abundance of *Bacteroidetes*, especially the genera of *Prevotella* and *Xylanbacter* (De Filippo et al., 2010; Trompette et al., 2014). For instance, *B. thetaiotaomicron* is regarded as a glycan-degrading generalist, which can degrade complex glycan of plant wall, animal tissue, and host mucin (Koropatkin et al., 2012). The change in the ratio of *Firmicutes* and *Bacteroidetes* in FCB group might be due to the glycan composition in FCB. Addition to *Firmicutes* and *Bacteroidetes* phylum, there were 13 other phylum detected among the 3 dietary treatments, including *Spirochaetae, Proteobacteria, Tenericutes, Lentisphaerae, Actinobacteria, SHA-109, Cyanobacteria, Fibrobacteres, Saccharibacteria, unclassified_k_norank, Synergistetes, Elusimicrobia*, and *Chlamydiae*. Compared to a previous study on gut microbiota composition in different age of pigs (Niu et al., 2015), wherein the *SHA-109, Saccharibacteria, unclassified_k_norank, and Elusimicrobia* were unique phylum, and this might be associated with different experiment diets between the studies. Specifically, *Fibrobacteres* phylum was identified by gene sequencing, albeit in a small proportion. The genus *Fibrobacter* belongs to the poorly defined *Fibrobacteres* phylum, which was regarded as *Spirochetes* or *Bacteroidetes*. In fact, the *Fibrobacteres* phylum is characterized by high cellulolytic activity and capable of degrading refractory plant structural polysaccharides and they play a prominent function in fiber-digesting and energy metabolism in gastrointestinal tract (Qi et al., 2005). In the rumen, the genus *Fibrobacter* is responsible for the degradation of dietary fiber and polysaccharides. The increased abundance of *Fibrobacter* resulted in more sugars and SCFAs in gastrointestinal tract of dairy calves (Deng et al., 2017). Moreover, *Fibrobacteres* was observed in different ages of pig; however, diet composition and fiber components were not mentioned (Niu et al., 2015). The mechanistic link between *Fibrobacteres* and polysaccharide metabolism needs further study.

Herein, the relative abundance of the top 30 bacterial communities at the genus level was shown in a heat map. *Clostridium* has been associated with dietary fiber metabolism previously (Niu et al., 2015). *Lachnospiraceae* and *Rumininococcaceae* are two dominant bacteria from *Clostridia* among the 3 dietary treatments and are regarded as fibrolytic specialists of commensal bacteria to degrade the complex plant material involved in activating carbohydrate enzymes, sugar transport, and metabolic pathways. Therefore, *Lachnospiraceae* and *Rumininococcaceae* are associated with the maintenance of gut health (Biddle et al., 2013). In this study, the CB group showed the greatest abundance of genus *Lachnospiraceae_NK4A136_group* and *Lachnospiraceae_XPB1014_group*. This was attributed to the dietary inclusion of CB which can stimulate fibrolytic bacteria to degrade the complex plant bran of recalcitrant substrate (Brulc et al., 2009). In contrast, non-adherent *Bacteriodes* sp. and *Bifidobacterium* sp. ferment easily hydrolysable starch (Cameron et al., 2014). Pigs fed CB treatment showed distinctly more bacteria in the *Lachnospiraceae* and *Rumininococcaceae* families of the *Firmicutes* phylum, while *Prevotellaceae* of the *Bacteroidetes* phylum was more abundant in the FCB group. The relative abundance of *Lactobacillus* and *Anaerovibrio* were higher in both CB and FCB groups compared to CON group. These genera are able to utilize glycerol and have been associated with propionate and butyrate production (Minuti et al., 2015). The result was supported by a previous study where insoluble dietary fiber extracted from CB stimulated the growth of beneficial bacteria in the gut of health individuals (Hsu et al., 2004). The *Prevotellaceae* family within *Bacteroidetes* phylum are involved in glucose metabolism (Heinritz et al., 2016) and the relative abundance of *norank_f_Prevotellaceae* was significantly higher in FCB group compared to the other 2 groups, consistent with the increased levels of soluble dietary fiber and glucose in FCB. *Rikenellaceae* are commensal bacteria in the gut in relatively small proportion, which can thrive on high-fat diets and are enriched in gut microbiota of the obese. A study reported that the abundance of *Rikenellaceae_RC9_gut_group* increased with high concentration of dietary protein in pigs (Fan et al., 2017a,b). In our study, the proportion of *Rikenellaceae_RC9_gut_group* decreased in CB and FCB treatments compared to the CON group, which suggested that *Rikenellaceae_RC9_gut_group* have an impact on carbohydrate and lipid metabolism. The alterations in microbial populations with the inclusion of CB and FCB are consistent with the relative fermentable substrate composition of feedstuff.

The end-product of bacterial metabolism plays an important role in shaping the bacterial community. Bacterial metabolite production is dependent on the available substrates, but in general, most end-products detectable in feces are acetate, propionate, and butyrate (Huang et al., 2015). Fermentation of different carbohydrate substrates by bacterial consortia normally results in variations of SCFAs production. Many colonic bacteria produce lactate as a fermentation product, but the concentration of lactate was <3–5 mM in fecal sample (Duncan et al., 2007). Lactic acid was not detected in the majority of fecal samples in our study. Lactate-utilizing bacteria includes *Eubacterium hallii* and *Anaerostipes caccae*, which can be detected in fecal samples from healthy individuals (Duncan et al., 2004). The species of *E. hallii* and *A. caccae* were identified by 16S rRNA sequencing in the present study, but their abundance were low and not affected by dietary treatments. Butyrate as one of the important bacterial metabolites that can provide energy for colonic epithelium, and also plays key functions in protection against colonic diseases (Koh et al., 2016). *Eubacterium rectale* and *Roseburia* species are two important groups of butyrate-producing bacteria. In the present study, the abundance of *Roseburia* was not influenced by dietary treatments, and a population of *E. rectale* was not observed in fecal samples. There were few changes on gut bacteria related to SCFAs production, and this observation was consistent with the stable SCFA concentration among the treatment groups.

In conclusion, diets containing CB and FCB did not impact growth performance or nutrient digestibility in finishing pigs. Gut microbiota composition was affected slightly by dietary treatments, as the bacterial richness and diversity in FCB was decreased compared to control and CB groups. Moreover, the relative abundance of bacteria related to cellulolytic degradation was higher in CB fed pigs; whereas FCB fed pigs had greater proportions of starch-fermenting bacteria. The differences in bacterial community between CB and FCB inclusion might be due to the alteration on chemical component of polysaccharides via *in vitro* fermentation. Thus, the underlying mechanisms on how the chemical characterizations of corn bran components influence the bacteria related to polysaccharide and lipid metabolism still needs further investigation.

## Author contributions

XM, and WL: Designed the experiments; PL, JinZ, PG, and ZG: Performed the experiments; LL and CW: Analyzed the data; PL: Wrote the manuscript, which was edited by CL, LJ, SQ, JieZ, and XM. XM: Resourced the project. All authors read and approved the final manuscript.

### Conflict of interest statement

The authors declare that the research was conducted in the absence of any commercial or financial relationships that could be construed as a potential conflict of interest.

## Abbreviations

ADFI: average daily feed intake
ADG: average daily gain
AIA: acid insoluble ash
ANOVA: one-way analysis of variance
CB: corn bran
CON: control
CP: crude protein
DM: dry matter
EE: ether extract
FCB: fermented corn bran
FCR: feed conversion ratio
GE: gross energy
MNB: mixed nutrient broth
MRS: de Man, rogosa and sharpe
OM: organic matter
OTUs: operational taxonomic units
SCFAs: short-chain fatty acids
TDF: total dietary fiber
YDP: yeast peptone dextrose.
