# Supplementary material for: Dietary Corn Bran Fermented by Bacillus subtilis MA139 Decreased Gut Cellulolytic Bacteria and Microbiota Diversity in Finishing Pigs
Source: Front Cell Infect Microbiol. 2017 Dec 22;7:526. doi: 10.3389/fcimb.2017.00526 (PMC5744180; doi:10.3389/fcimb.2017.00526)
Supplement: Supplementary file 1 [file Table1.DOCX]

**Supplemental Table 1** The composition of experimental diets and nutritional values (as-fed basis)

|  | Dietary treatments | | |  |
| --- | --- | --- | --- | --- |
| Item | CON | CB | FCB |  |
| Ingredients, % |  |  |  |  |
| Corn | 75.20 | 65.90 | 65.80 |  |
| Soybean meal | 22.00 | 20.50 | 21.50 |  |
| Corn bran | **-** | **10.00** | **-** |  |
| Fermented corn bran | **-** | **-** | **10.00** |  |
| Soybean oil | **-** | 0.10 | 0.10 |  |
| Dicalcium phosphate | 0.65 | 0.60 | 0.60 |  |
| Limestone | 0.85 | 0.70 | 0.80 |  |
| Salt | 0.35 | 0.30 | 0.30 |  |
| Vitamin-mineral premix^1^ | 0.50 | 0.50 | 0.50 |  |
| Methionine | 0.03 | 0.02 | 0.03 |  |
| Threonine | 0.06 | 0.04 | 0.06 |  |
| Tryptophan | 0.04 | 0.03 | 0.04 |  |
| Valine | 0.02 | 0.02 | 0.02 |  |
| Total | 100.00 | 100.00 | 100.00 |  |
| Nutritional level | | | | |
| ME, MJ/kg | 14.02 | 14.02 | 14.02 |  |
| CP, % | 15.35 | 15.35 | 15.35 |  |
| SID Lysine, % | 0.88 | 0.88 | 0.88 |  |
| SID Methionine, % | 0.27 | 0.27 | 0.27 |  |
| SID Threonine, % | 0.54 | 0.54 | 0.54 |  |
| SID Tryptophan, % | 0.17 | 0.17 | 0.17 |  |
| SID Valine, % | 0.58 | 0.58 | 0.58 |  |

^1^Premix provided the following per kg of complete diet for finishing pigs: vitamin A, 5,512 IU; vitamin D3, 2,200 IU; vitamin E, 30 IU; vitamin K3, 2.2 mg; vitamin B12, 27.6 μg; riboflavin, 4.0 mg; pantothenic acid, 14.0 mg; niacin, 30.0 mg; choline chloride, 400.0 mg; folacin, 0.7 mg; thiamine 1.5 mg; pyridoxine 3.0 mg; biotin, 44.0 μg; Mn (MnO), 40.0 mg; Fe (FeSO_4_•H_2_O), 75.0 mg; Zn (ZnO), 75.0 mg; Cu (CuSO_4_•5H_2_O), 100.0 mg; I (KI), 0.3 mg; Se (Na_2_SeO_3_), 0.3 mg. CON, control group; CB, corn bran; FCB, fermented corn bran, SID, standardized ileal digestible.
