# Supplementary material for: Dietary Corn Bran Fermented by Bacillus subtilis MA139 Decreased Gut Cellulolytic Bacteria and Microbiota Diversity in Finishing Pigs
Source: Front Cell Infect Microbiol. 2017 Dec 22;7:526. doi: 10.3389/fcimb.2017.00526 (PMC5744180; doi:10.3389/fcimb.2017.00526)
Supplement: Supplementary file 2 [file Table2.DOCX]

**Supplemental Table 2** The relative abundance of bacterial communities at the phylum level in finishing pigs fed different corn bran inclusions^1^

| Taxa | Dietary treatments | | |  |
| --- | --- | --- | --- | --- |
| Phylum | CON (%) | CB (%) | FCB (%) | *P*-value |
| Firmicutes | 61.39 | 62.96 | 56.05 | 0.186 |
| Bacteroidetes | 34.08 | 31.65 | 39.23 | 0.149 |
| Spirochaetae | 1.74 | 2.29 | 1.46 | 0.483 |
| Proteobacteria | 1.27 | 1.44 | 1.53 | 0.437 |
| Tenericutes | 0.49 | 0.52 | 0.33 | 0.534 |
| Lentisphaerae | 0.33 | 0.25 | 0.28 | 0.776 |
| Actinobacteria | 0.30 | 0.30 | 0.22 | 0.496 |
| SHA-109 | 0.09 | 0.20 | 0.53 | 0.159 |
| Cyanobacteria | 0.20 | 0.15 | 0.21 | 0.526 |
| Fibrobacteres | 0.04 | 0.13 | 0.09 | 0.078 |
| Saccharibacteria | 0.04 | 0.07 | 0.03 | 0.472 |
| unclassified_k_norank | 0.02 | 0.01 | 0.01 | 0.315 |
| Synergistetes | 0.01 | 0.02 | < 0.01 | 0.035 |
| Elusimicrobia | < 0.01 | < 0.01 | 0.01 | 0.544 |
| Chlamydiae | < 0.01 | < 0.01 | < 0.01 | 0.767 |

^1^Fecal samples from 7 pigs per treatment were performed for 16S rRNA gene amplicon sequencing analysis in a 21 d feeding trial. The results were analyzed by Kruskal-Wallis H test, and data were presented as mean percentage. CON, control group; CB, corn bran; FCB, fermented corn bran.
