# Supplementary material for: Dietary Corn Bran Fermented by Bacillus subtilis MA139 Decreased Gut Cellulolytic Bacteria and Microbiota Diversity in Finishing Pigs
Source: Front Cell Infect Microbiol. 2017 Dec 22;7:526. doi: 10.3389/fcimb.2017.00526 (PMC5744180; doi:10.3389/fcimb.2017.00526)
Supplement: Supplementary file 3 [file Table3.DOCX]

**Supplemental Table 3** The relative abundance of bacterial communities at the family level in finishing pigs fed different corn bran inclusions ^1^

| Taxa | | Dietary treatments | | |  |
| --- | --- | --- | --- | --- | --- |
| Phylum | Family | CON (%) | CB (%) | FCB (%) | *P*-value |
| Firmicutes | Clostridiaceae_1 | 16.56 | 16.14 | 12.90 | 0.754 |
|  | Veillonellaceae | 7.76 | 8.65 | 15.23 | 0.293 |
|  | Ruminococcaceae | 11.05 | 9.47 | 6.24 | 0.079 |
|  | Lachnospiraceae | 7.83 | 10.78 | 7.21 | 0.107 |
|  | Streptococcaceae | 6.08 | 6.32 | 4.54 | 0.324 |
|  | Peptostreptococcaceae | 4.70 | 4.55 | 4.09 | 0.698 |
|  | Lactobacillaceae | 1.91 | 2.71 | 3.18 | 0.498 |
|  | Christensenellaceae | 2.64 | 1.83 | 0.52 | 0.252 |
|  | Acidaminococcaceae | 1.04 | 0.78 | 0.98 | 0.789 |
|  | Erysipelotrichaceae | 0.99 | 0.91 | 0.63 | 0.168 |
|  | Family_XIII | 0.40 | 0.36 | 0.22 | 0.085 |
|  | Clostridiales_vadinBB60_group | 0.22 | 0.17 | 0.12 | 0.830 |
|  | Peptococcaceae | 0.11 | 0.12 | 0.07 | 0.344 |
| Bacteroidetes | Bacteroidales_S24-7_group | 3.93 | 5.20 | 3.11 | 0.355 |
|  | Rikenellaceae | 2.62 | 1.91 | 1.44 | 0.074 |
|  | Prevotellaceae | 25.81 | 23.06 | 33.77 | 0.094 |
|  | Porphyromonadaceae | 1.07 | 0.80 | 0.41 | 0.103 |
|  | unclassified_o_Bacteroidales | 0.29 | 0.19 | 0.15 | 0.274 |
|  | norank_o_Bacteroidales | 0.08 | 0.04 | 0.21 | 0.332 |
|  | Bacteroidaceae | 0.12 | 0.13 | 0.06 | 0.770 |
|  | Bacteroidales_RF16_group | 0.09 | 0.15 | 0.03 | 0.529 |
|  | Bacteroidales_BS11_gut_group | 0.02 | 0.15 | 0.01 | 0.461 |
| Proteobacteria | Succinivibrionaceae | 0.70 | 0.47 | 0.65 | 0.757 |
|  | GR-WP33-58 | 0.11 | 0.43 | 0.45 | 0.078 |
|  | Campylobacteraceae | 0.14 | 0.20 | 0.12 | 0.192 |
|  | Desulfovibrionaceae | 0.18 | 0.16 | 0.11 | 0.291 |
| Spirochaetae | Spirochaetaceae | 1.74 | 2.29 | 1.46 | 0.483 |
| SHA-109 | norank_p_SHA-109 | 0.09 | 0.20 | 0.53 | 0.159 |
| Tenericutes | norank_o_Mollicutes_RF9 | 0.49 | 0.52 | 0.33 | 0.511 |
| Actinobacteria | Coriobacteriaceae | 0.27 | 0.29 | 0.21 | 0.552 |
| Lentisphaerae | norank_c_Lentisphaerae_RFP12_gut_group | 0.30 | 0.19 | 0.26 | 0.438 |
| Cyanobacteria | norank_o_Gastranaerophilales | 0.20 | 0.15 | 0.21 | 0.526 |
| Fibrobacteres | Fibrobacteraceae | 0.04 | 0.13 | 0.09 | 0.077 |

^1^Fecal samples from 7 pigs per treatment were performed for 16S rRNA gene amplicon sequencing analysis in a 21 d feeding trial. The results were analyzed by Kruskal-Wallis H test, and data were presented as mean percentage. CON, control group; CB, corn bran; FCB, fermented corn bran.
