# Supplementary material for: Dietary Corn Bran Fermented by Bacillus subtilis MA139 Decreased Gut Cellulolytic Bacteria and Microbiota Diversity in Finishing Pigs
Source: Front Cell Infect Microbiol. 2017 Dec 22;7:526. doi: 10.3389/fcimb.2017.00526 (PMC5744180; doi:10.3389/fcimb.2017.00526)
Supplement: Supplementary file 4 [file Table4.DOCX]

**Supplemental Table 4** The relative abundance of the top 30 bacterial communities at the genus level in finishing pigs fed different corn bran inclusions^1^

| Taxa |  |  |  |  | Dietary treatments | | |  |
| --- | --- | --- | --- | --- | --- | --- | --- | --- |
| Phylum | Class | Order | Family | Genus | CON (%) | CB (%) | FCB (%) | *P*-value |
| Firmicutes | Clostridia | Clostridiales | Clostridiaceae_1 | Clostridium_sensu_stricto_1 | 16.47 | 16.04 | 12.86 | 0.746 |
|  |  |  | Peptostreptococcaceae | Terrisporobacter | 3.84 | 3.69 | 3.38 | 0.797 |
|  |  |  | Lachnospiraceae | Lachnospiraceae_NK4A136_group | 0.92 | 1.29 | 0.26 | 0.006 |
|  |  |  |  | unclassified_f_Lachnospiraceae | 1.11 | 1.58 | 1.07 | 0.112 |
|  |  |  |  | Pseudobutyrivibrio | 1.21 | 1.00 | 1.50 | 0.875 |
|  |  |  |  | Lachnospiraceae_XPB1014_group | 0.82 | 2.32 | 0.29 | 0.157 |
|  |  |  |  | Roseburia | 0.72 | 1.25 | 1.29 | 0.879 |
|  |  |  | Ruminococcaceae; | Ruminococcaceae_NK4A214_group | 0.91 | 0.81 | 0.57 | 0.366 |
|  |  |  |  | Ruminococcus_1 | 1.17 | 1.05 | 0.68 | 0.631 |
|  |  |  |  | Ruminococcaceae_UCG-002 | 1.59 | 1.18 | 0.83 | 0.350 |
|  |  |  |  | Ruminococcaceae_UCG-005 | 2.27 | 2.18 | 1.11 | 0.095 |
|  |  |  | Christensenellaceae | Christensenellaceae_R-7_group | 2.64 | 1.83 | 0.52 | 0.251 |
|  | Bacilli | Lactobacillales | Streptococcaceae | Streptococcus | 6.07 | 6.31 | 4.54 | 0.641 |
|  |  |  | Lactobacillaceae | Lactobacillus | 1.91 | 2.71 | 3.18 | 0.498 |
|  | Negativicutes | Selenomonadales | Veillonellaceae | Anaerovibrio | 0.85 | 2.09 | 2.72 | 0.577 |
|  |  |  |  | Megasphaera | 2.51 | 2.95 | 5.10 | 0.152 |
|  |  |  |  | Selenomonas | 2.82 | 1.69 | 4.63 | 0.521 |
| Bacteroidetes | Bacteroidia | Bacteroidales | Bacteroidales_S24-7_group | norank_f_Bacteroidales_S24-7_group | 3.93 | 5.20 | 3.11 | 0.355 |
|  |  |  | Prevotellaceae | Prevotellaceae_NK3B31_group | 8.23 | 7.44 | 9.61 | 0.891 |
|  |  |  |  | Prevotella_1 | 2.87 | 3.44 | 3.77 | 0.507 |
|  |  |  |  | Prevotella_2 | 1.18 | 1.00 | 1.08 | 0.837 |
|  |  |  |  | Prevotella_7 | 1.09 | 0.49 | 1.20 | 0.069 |
|  |  |  |  | Prevotella_9 | 6.73 | 6.71 | 12.37 | 0.328 |
|  |  |  |  | Prevotellaceae_UCG-003 | 1.86 | 1.14 | 1.50 | 0.610 |
|  |  |  |  | Alloprevotella | 1.84 | 1.04 | 1.49 | 0.314 |
|  |  |  |  | unclassified_f_Prevotellaceae | 1.07 | 0.48 | 0.86 | 0.244 |
|  |  |  |  | norank_f_Prevotellaceae | 0.69 | 0.64 | 1.47 | 0.039 |
|  |  |  | Rikenellaceae | Rikenellaceae_RC9_gut_group | 2.24 | 1.61 | 1.17 | 0.048 |
| Firmicutes | Negativicutes | Selenomonadales | Veillonellaceae | unclassified_f_Veillonellaceae | 0.80 | 0.73 | 1.09 | 0.698 |
| Spirochaetae | Spirochaetes | Spirochaetales | Spirochaetaceae | Treponema_2 | 1.55 | 2.18 | 1.23 | 0.437 |

^1^Fecal samples from 7 pigs per treatment were performed for 16S rRNA gene amplicon sequencing analysis in a 21 d feeding trial. The results were analyzed by Kruskal-Wallis H test, and data were presented as mean percentage. CON, control group; CB, corn bran; FCB, fermented corn bran.
